# Job insecurity during recessions: effects on survivors’ work stress

**DOI:** 10.1186/1471-2458-13-929

**Published:** 2013-10-06

**Authors:** Sepideh Modrek, Mark R Cullen

**Affiliations:** 1 General Medical Disciplines, Stanford University School of Medicine, Palo Alto, CA, USA

**Keywords:** Economic recession, Downsizing, Work stress

## Abstract

**Background:**

Previous studies show a variety of negative health consequences for the remaining workforce after downsizing events. This study examined self-reported work stress from 2009–2012 in the context of a large multi-site aluminum manufacturing company that underwent severe downsizing in 2009.

**Methods:**

This study examined the association between work stress and working at a work site that underwent severe downsizing. We assessed the level of downsizing across thirty plants in 2009 and categorized seven as having undergone severe downsizing. We linked plant-level downsizing information to individual workers’ responses to an annual work engagement survey, which included three work stress questions. From 2009 to 2012 over 14, 000 employees were asked about their experience of work stress. Though the surveys were anonymous, the surveys captured employees’ demographic and employment characteristic as well as plant location. We used hierarchical logistic regressions to compare responses of workers at severely downsized plants to workers at all other plant while controlling for demographic and plant characteristics. Responses to the work stress questions and one control question were examined.

**Results:**

In all yearly surveys salaried workers consistently reported having more work stress than hourly workers. There was no differential in work stress for workers at severely downsized plants in 2009. In 2010 to 2012, salaried workers who remained at severely downsized plants reported significantly higher work stress than salaried workers at all other plants across multiple work stress questions. Examination of the 2006 survey confirmed that there were no pre-existing differences in work stress among salaried employees working at plants that would eventually experience severe downsizing. In addition, there was no difference in responses to the control question at severely downsized plants.

**Conclusion:**

Salaried workers at plants with high layoffs experienced more work stress after 2009 than their counterparts at non-high layoff plants. Increased work stress is important to monitor and may be a mediating pathway through which the external economic environment leads to adverse health outcomes.

## Background

Throughout the economic recession of 2007–2009 organizations reduced personnel through downsizing and layoffs leading to widespread increase in job insecurity even for those who remain employed [1]. Previous research has found job insecurity to be associated with worse mental and physical health outcomes for the remaining workforce, hereafter referred to as survivors [2-4]. Many of these studies examined perceptions of work insecurity (as opposed to externally measured downsizing) and were undertaken in non-recessionary periods. During recessions when labor markets are weak and other work opportunities may be more limited, the sense of job insecurity may be particularly harmful. According to the American Life Panel survey conducted from 2008–2010, workers consistently overestimated their probability of losing their job in the upcoming year, suggesting persistent job insecurity was a prominent issue during the recent recession [5].

In addition to job insecurity, downsizing is likely to be accompanied by increased workload for the surviving workers. Studies of health care workers after downsizing suggest there is an association between downsizing and greater workload [6]. Both greater workload and job insecurity may in turn cause work stress [7], which previous studies have shown to have detrimental effects on health. Both stress at work and job strain, a related construct where employees have high work demands and low control, have been found to be associated with coronary heart disease [8,9], metabolic syndrome [10,11], and elevated blood pressure [12-14].

In this study, we examined workers’ self-reported work stress at a large multi-site, geographically diverse, aluminum-manufacturing company, Alcoa. Our study leveraged large annual company wide work engagement surveys from 2009–2012, which included data during the height of and after the large layoffs in 2009. We exploited the variation in the timing and intensity of layoffs at thirty Alcoa plants to categorize each plant as either having high layoffs or not. We then examined the relationship between plant-level intensity of downsizing in 2009 and individual-level perceptions of work stress for surviving workers from 2009–2012.

### Study context

As a consequence of rapidly declining demand for products in the construction, aerospace and automotive industries, Alcoa was forced to cut production broadly leading to layoffs at every level of the organization in 2009 [15]. However, not all worksites experienced similar levels layoffs. Some worksites let go of as much as 40% of their workforce while others lost only 5% — a turnover similar to that of normal times. We used the plant level variation in the severity of layoff to categorize plants by the intensity of the 2009 downsizing and evaluated perceptions of work stress for the remaining workforce from 2009 to 2012.

The outcome of interest, work stress, has been collected yearly through an anonymous survey of work engagement since 2006. The yearly surveys include three questions that deal specifically with work stress with identical wording and scales in all surveys. The survey is administered over a 20-day period between September and October of each year and employees are given time at work to complete it. Each survey is de-identified, but gender, race, tenure, employee type (hourly vs. salaried) and plant location are captured. The company encouraged employees to respond, providing them with paid time to respond to the survey, which contributed to the high response rates—exceeding 80% overall since 2008.

## Methods

### Data

#### Independent variable of interest: external measure of job insecurity

To create a measure of downsizing, we used personnel data. For thirty fully functional US plants with greater than 100 employees in 2008, we calculated the proportion of the workforces that was laid off in 2009. For these same plants we noted dates in 2009 where more than forty workers had their contract of employment terminated on the same date and classify these dates as mass layoff events. The downsizing measure was then defined by two criteria: 1) the proportional change in size of the workforce at the plant in 2009 and 2) having experienced a mass termination event. Worksites with 20-40% reductions in the workforce and a mass termination event were categorized as high layoffs plants. Using this definition, seven plants were considered high layoff plants. The remaining twenty-three plants served as control plants though workers at control plants experienced some downsizing as well. These cut offs are similar to those used in previous studies of examining downsizing events [16,17].

#### Individual-level self-reported work stress

Alcoa conducts an anonymous annual survey of work engagement administered through a third party vendor. The company then receives department level scores for the 32-question survey. At the request of the research team the third party vendor has included three validated work stress questions derived from previous surveys of work organization [18]. Workers were asked how much they agreed or disagreed, on a 5-point Likert scale, to the follow questions: 1) I find my work stressful, 2) I find that I am worn out at the end of the day, and 3) I find that work issues frequently remain on my mind after hours. In our analyses responses to each question were dichotomized. Workers who reported that they agree or strongly agree with each statement were considered to have work stress in the analysis to follow and each question is analyzed individually (though the Cronbach internal consistently score for the three questions ranges from 0.78-0.84).

**Figure 1:**
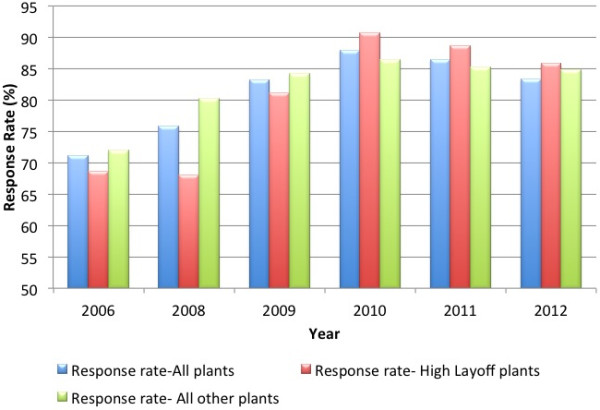
Work engagement survey response rate, by year and level of layoffs in 2009.

Figure 1 presents the response rate by year and plant type for the overall survey. The response rate was consistently over 70%, but response rates were substantially lower in 2006 (71%) and 2008 (75%). Data from the 2007 survey were not examined because plant identifiers could not be ascertained. From 2009 to 2012, response rates were relatively stable ranging from 83-87%. We excluded the 2008 surveys in the regression analyses and focused on the 2009–2012 surveys because 1) the response rate was lower and there were substantial differences in response rates by plant type, 2) the survey was conducted during the peak of the 2008 stock market crash and therefore responses may include ambient stressors and acute financial stressors outside of work, and 3) the layoffs in 2009 changed the composition of the work force dramatically from 2008. In supplemental analyses we examined the 2006 data to assess whether there were any pre-existing differences in reported work stress in plants that would eventually have high layoffs three years later. We present these analyses separately because the composition of the workforces who responded to the survey was different in 2006.

The responses to these surveys were anonymous. Though many of the same workers replied to the 2009–2012 surveys, we can not link individual responses from year to year, which precluded us from doing individual-level longitudinal analyses, i.e. panels. In the regression analyses detailed below, we examined the responses to three work stress questions controlling for basic demographic characteristics collected in each survey year. These characteristics included gender, race, hourly/salary status, and tenure. In addition we used the change in county-level unemployment rate in each prior year (compiled from Bureau of Labor Statistics data http://www.bls.gov) as an additional area-level control to capture the conditions of the local labor markets.

### Statistical methods

For perceived work stress, the three work stress questions were individually examined. We first described patterns of responses to each of the work stress questions over time, from 2006 and 2008–2012 by demographic group. We employed hierarchical logistic regressions to assess the association of an employee working in a plant categorized as having a high level of layoffs in 2009 and reporting work stress. Since each observation corresponds to one worker’s survey responses within a plant, the hierarchical model accounts for clustering of survey responses within a plant. For each of the three work stress questions we used the logistic function to model the binary outcomes. We estimated the following models:

Model 1

$$\begin{array}{c} {\text{Work}\;\text{Stress}}_{p,i}=F_{\text{logistic}}\left(\alpha +\beta 1\left({\text{Sex}}_{i}\right)+\beta 2\left({\text{Race}}_{i}\right)\right)\\ \;+\beta 3\left({\text{Tenure}}_{i}\right)+\beta 4\left({\text{Union}}_{p}\right)\\ \;+\beta 5\left(\Delta {\text{Area}\;\text{Unemployment}}_{p}\right)\\ \;+\beta 6\left({\text{Salary}}_{i}\right)+\beta 7\left({\text{High}\;\text{Layoff}}_{p}\right)\\ \;\left(+\gamma_{\mathrm{pi}}\right) \end{array}$$

Model 2

$$\begin{array}{l} {\text{Work}\;\text{Stress}}_{p,i}=F_{\text{logistic}}\left(\alpha +\beta 1\left({\text{Sex}}_{i}\right)+\beta 2\left({\text{Race}}_{i}\right)\right)\\ \;+\beta 3\left({\text{Tenure}}_{i}\right)+\beta 4\left({\text{Union}}_{p}\right)\\ \;+\beta 5\left(\Delta {\text{Area}\;\text{Unemployment}}_{p}\right)\\ \;+\beta 6\left({\text{Salary}}_{i}\right)+\beta 7\left({\text{High}\;\text{Layoff}}_{p}\right)\\ \;\left(+\beta 8\left({\text{Salary}}_{i}*{\text{High}\;\text{Layoff}}_{p}\right)+\gamma_{\mathrm{pi}}\right) \end{array}$$

Model 2 is similar to Model 1 but allows for differential effects by worker type of working at a high layoff plant. Since each observation corresponds to one worker’s survey responses within a plant, we modeled the overall error term, γ_pi_ = α_i_ + ϵ_pi_, using a random-effects at the plant-level to account for the interdependence of the observations within plants. In addition, we included all available individual-level covariate controls as well as a control for changes in county-level unemployment rates in each preceding year.

In addition to the three work stress questions, we also examined a fourth question throughout, “I am proud to work at Alcoa”. Responses to this pride question were not correlated to the work stress question and are meant served as a control question in our analyses. The analysis of control question attempted to capture any *systematic* bias in employee responses. For example, there might have been some bias towards positive responses about the company in general after the 2009 downsizing from workers to exude an optimistic impression towards management, but these biases would lead to an underestimate of the effect of downsizing. On the other hand, if there were residual anger or disappointment towards the company for layoffs, then we would expect lower reports of pride in the company at high layoff plants. Overall, we expected less *systematically* positive or negative responses to the work stress questions because they were added to the survey by the research team and were not used in the third party vendor’s assessment of work engagement.

In additional analyses we examined survey responses in the 2006 data. The composition of respondents to the 2006 survey was likely quite different than the respondents to the 2009–2012 surveys because of attrition, new hires and layoffs. Therefore, the two sets of analyses are not fully comparable. Nonetheless, the analysis of the 2006 data was conducted to assess pre-existing differences in work stress by plants that would eventually have high levels of layoffs. We also examined the control question in 2006.

### Institutional review

The Stanford University Institutional Review Board approved this study’s protocol, invoking the epidemiologic exemption waiving the requirement for individual consent.

## Results

### Main analyses

Table 1 provides descriptive statistics for the analytic sample. For each year in 2006 and 2009–2012 the mean proportion who agree with the work stress questions, the pride question, as well as the sample characteristics collected in the survey are presented. For each individual work stress item, 37-55% of the sample report work stress. While these proportions are relatively stable from 2009–2012, reports of work stress are much lower for all the questions in 2006. For the control pride question, there is a notable increase in the proportion of the sample that report being proud to work at Alcoa from 45% in 2006, to 57% in 2009 to 79% by 2012. The demographic characteristics of this sample are comparable to the entire Alcoa workforces; predominately white (70%), male (79%), hourly workers (70%) who have been working at Alcoa for at least 6 years (75%) for 2009–2012. The sample of survey respondents in 2006 include workers with shorter tenure, more salaried workers, and more workers from plants that would eventually experience high layoffs. Due to these differences, we examine the 2006 survey data separately.

**Table 1:** Outcome means and sample characteristics

	**Survey year**				
	**2006**	**2009**	**2010**	**2011**	**2012**
**Outcomes (% who agree or strongly agree)**					
I find my work stressful.	40%	50%	49%	46%	46%
I find that I am worn out at the end of the day.	44%	55%	55%	53%	53%
I find that work issues frequently remain on my mind after hours.	37%	44%	44%	43%	41%
I am proud to work at Alcoa.	45%	57%	64%	78%	79%
**Demographic controls**					
White	60%	70%	70%	70%	67%
Male	78%	79%	79%	79%	79%
Salary	30%	29%	25%	25%	25%
Tenure					
Less than 1 year	8%	1%	3%	9%	5%
1-2 years	13%	8%	4%	4%	10%
3-5 years	10%	17%	18%	15%	10%
6-10 years	19%	15%	17%	19%	20%
11-20 years	20%	26%	26%	24%	25%
Over 20 years	30%	33%	33%	30%	29%
Work at a plant with High Layoffs in 2009	39%	33%	34%	34%	34%
Observations	13980	14658	16144	15703	15667
Plants	30	30	30	30	30
Union plants	8	8	8	8	8
High layoff plants	7	7	7	7	7

Note: In this table the number of observations reflects the sample that responded to all the outcome questions. For any given individual outcome, the number of observations may be slightly higher.

For 2009–2012, the years of the main analyses, these demographic characteristics of the sample remain stable, though there is some difference in worker tenure over the four survey years. In 2009 less than 1% and in 2010 about 3% of workers report working at Alcoa for less than 1 year. There is some more hiring in 2011–2012 as is evident by the 5-8% of workers who report work at Alcoa for less than one year. Overall, most workers in the sample are long tenured employees. A large proportion of these workers, 34%, work at the 7 plants that had high layoffs in 2009 and 37% of them work at plants with union contracts for hourly workers.

**Figure 2:**
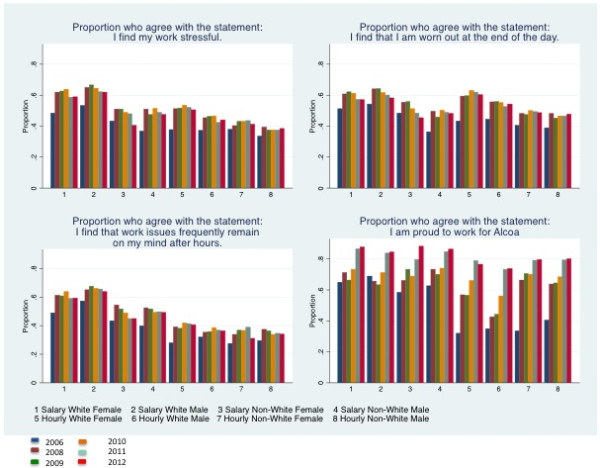
Work stress questions and control question 2008–2012 survey means for 2006 & 2008–2012 surveys, by demographic group.

In Figure 2 we present the proportion of employees who agree/disagree with the three work stress questions and the pride question in the 2006, and 2008 to 2012 surveys. For each question we aggregated the mean responses over three factors, gender, employee type and race. While the composition of survey respondents changed in each survey year (the 2006 survey was conducted prior to any knowledge of layoffs or the upcoming recession and includes many workers who would be leave Alcoa before 2009 or be laid off in 2009, the 2008 survey was conducted before large layoffs but at the peak of the stock market crash and includes may workers who would be laid off in 2009, the 2009 survey was conducted after several rounds of layoffs but before all layoffs were completed, the 2010 to 2012 surveys were conducted after much of the workforce had stabilized), the figure indicates that regardless of survey year salaried workers were more likely to report more work stress. The figure also shows that regardless of the demographic group, workers reported much lower work stress in 2006. In contrast, there is no evidence of a marked difference in the proportion of workers that agree or strongly agree with the work stress statements between 2008–2012. Figure 2 also shows large increases in the proportion of employees who report that they are proud to work at Alcoa in 2010–2012 as compared to 2006 or 2008–2009 across all demographic groups; the largest increase in reporting pride in working at Alcoa was among hourly workers from 2006 to 2008.

Table 2 reports the association between working at a high layoff plant, the three work stress questions and the pride question from 2009–2012, our main analyses. The table presents both Model 1 and 2 for each question. In column one and two, we examine responses to the question, I find my work stressful. In 2009, we find that while more salaried workers report that they find their work to be stressful overall as compared to hourly workers (OR = 2.14; 95% CI 1.95 - 2.34), there is no difference for workers who work at high layoff plants. In contrast by 2010, more salaried workers at high layoff plants report that they find their work to be stressful compared to salaried workers at non-high layoff plants (OR = 1.30; CI = 1.11-1.53). This differential is found again in 2011 and 2012 and its magnitude and significance remain quite stable. Results for the second work stress question, I find that I am worn out at the end of the day, are presented in columns three and four. We find that salaried workers reported being worn out more than hourly workers in all the survey years. In addition, there is no differential for salaried workers at high layoff plants compared to salaried workers at other plants in 2009 or 2010. However, by 2011 more salaried workers at high layoff plants reported being worn out than salaried workers at all other plants (OR = 1.26; CI 1.068 - 1.486). This differential is observed again in the 2012 survey with very similar significance and magnitude to those found in 2011. Results for the final work stress question, I find that work issues frequently remain on my mind after hours, are presented in column five and six. Salaried workers reported higher rates of having work remain on their minds in all years. For this question there is no significant differential for salaried workers who worked at high layoff plants in any of the four years. In column seven and eight, we examined a control question, one not related to the work stress question but asked in the same survey, I am proud to work at Alcoa. Salaried workers consistently reported higher rates of pride in working at Alcoa as compared to hourly workers. However, for this question there was no differential at high layoff plants.

**Table 2:** Association between working at a high layoff plant and the work stress questions and the pride question from 2009–2012, odd ratios and 95% confidence internals

	**I find my work stressful**		**I find that I am worn out at the end of the day**		**I find that work issues frequently remain on my mind after hours**		**I am proud to work at Alcoa**	
2009 Survey								
	*Model 1*	*Model 2*	*Model 1*	*Model 2*	*Model 1*	*Model 2*	*Model 1*	*Model 2*
High layoff plant	0.895	0.901	0.92	0.943	1.009	1.048	1.139	1.097
	[0.666 - 1.201]	[0.669 - 1.215]	[0.665 - 1.273]	[0.679 - 1.309]	[0.834 - 1.221]	[0.860 - 1.275]	[0.734 - 1.766]	[0.706 - 1.704]
Salary	2.118***	2.136***	1.383***	1.422***	3.062***	3.193***	1.812***	1.739***
	[1.963 - 2.285]	[1.948 - 2.343]	[1.282 - 1.491]	[1.297 - 1.558]	[2.838 - 3.304]	[2.911 - 3.502]	[1.675 - 1.961]	[1.583 - 1.909]
Salaried * high layoff plants		0.975		0.918		0.88		1.149
		[0.831 - 1.143]		[0.784 - 1.076]		[0.750 - 1.032]		[0.969 - 1.362]
Observations	14760	14760	14748	14748	14762	14762	14776	14776
Number of locations	30	30	30	30	30	30	30	30
2010 Survey								
	*Model 1*	*Model 2*	*Model 1*	*Model 2*	*Model 1*	*Model 2*	*Model 1*	*Model 2*
High layoff plant	0.935	0.876	0.928	0.906	1.067	1.051	1.058	1.037
	[0.678 - 1.289]	[0.634 - 1.212]	[0.659 - 1.305]	[0.642 - 1.278]	[0.832 - 1.367]	[0.818 - 1.351]	[0.663 - 1.687]	[0.650 - 1.655]
Salary	1.929***	1.773***	1.230***	1.193***	2.656***	2.606***	1.763***	1.712***
	[1.789 - 2.081]	[1.619 - 1.943]	[1.141 - 1.326]	[1.089 - 1.306]	[2.463 - 2.865]	[2.379 - 2.856]	[1.624 - 1.915]	[1.550 - 1.890]
Salaried * high layoff plants		1.301***		1.101		1.061		1.097
		[1.107 - 1.528]		[0.940 - 1.291]		[0.904 - 1.246]		[0.921 - 1.306]
Observations	16185	16185	16187	16187	16184	16184	16169	16169
Number of locations	30	30	30	30	30	30	30	30
2011 Survey								
	*Model 1*	*Model 2*	*Model 1*	*Model 2*	*Model 1*	*Model 2*	*Model 1*	*Model 2*
High layoff plant	0.999	0.927	0.97	0.919	1.048	1.022	1.122	1.114
	[0.660 - 1.510]	[0.613 - 1.403]	[0.628 - 1.497]	[0.595 - 1.421]	[0.755 - 1.453]	[0.735 - 1.420]	[0.695 - 1.813]	[0.688 - 1.802]
Salary	1.974***	1.788***	1.240***	1.154***	2.685***	2.598***	1.859***	1.835***
	[1.826 - 2.134]	[1.628 - 1.962]	[1.148 - 1.339]	[1.052 - 1.266]	[2.484 - 2.902]	[2.367 - 2.853]	[1.680 - 2.058]	[1.622 - 2.075]
Salaried * high layoff plants		1.376***		1.260***		1.111		1.042
		[1.164 - 1.627]		[1.068 - 1.486]		[0.940 - 1.312]		[0.841 - 1.290]
Observations	15730	15730	15732	15732	15731	15731	15726	15726
Number of locations	30	30	30	30	30	30	30	30
2012 Survey								
	*Model 1*	*Model 2*	*Model 1*	*Model 2*	*Model 1*	*Model 2*	*Model 1*	*Model 2*
High layoff plant	1.002	0.941	0.979	0.934	1.093	1.078	1.032	1.019
	[0.671 - 1.496]	[0.630 - 1.405]	[0.624 - 1.537]	[0.594 - 1.466]	[0.783 - 1.527]	[0.771 - 1.509]	[0.608 - 1.750]	[0.600 - 1.732]
Salary	1.863***	1.712***	1.141***	1.068	2.701***	2.653***	2.000***	1.956***
	[1.723 - 2.016]	[1.558 - 1.881]	[1.055 - 1.233]	[0.972 - 1.173]	[2.496 - 2.923]	[2.413 - 2.918]	[1.799 - 2.224]	[1.722 - 2.222]
Salaried * high layoff plants		1.310***		1.231**		1.059		1.073
		[1.108 - 1.549]		[1.043 - 1.452]		[0.895 - 1.252]		[0.856 - 1.343]
Observations	15715	15715	15711	15711	15714	15714	15703	15703
Number of locations	30	30	30	30	30	30	30	30

95% Confidence intervals are in brackets.

Asterisk indicate significance level of estimated coefficient: *** p < 0.01, ** p < 0.05, * p < 0.1.

Note: Both model 1 & 2 include controls for worker’s tenure, worker’s race, worker’s gender, whether the worker was employed at a union plant, and the change in the prior year’s unemployment rate in the country of the plant. Model 2 incudes an additional interaction term between working at a high layoff plant and worker type to allow for a differential effect of working at a high layoff plant by worker type on stress outcomes.

### Analyses of pre-existing trends

Table 3 reports the association between the same questions as above and working at a plant that would eventually have high layoffs in three years. Many respondents in the 2006 sample will have been laid off in the 2009 downsizing events. Nonetheless, the analysis was necessary to show whether there were any differences in reports of stress well before the onset of the recession and layoffs to come.

**Table 3:** Association between working at a high layoff plant and the work stress questions and the pride question from 2006, odd ratios and 95% confidence intervals

	**I find my work stressful**		**I find that I am worn out at the end of the day**		**I find that work issues frequently remain on my mind after hours**		**I am proud to work at Alcoa**	
2006 Survey								
	*Model 1*	*Model 2*	*Model 1*	*Model 2*	*Model 1*	*Model 2*	*Model 1*	*Model 2*
High layoff plant	1.202	1.189	1.225	1.207	1.194	1.192	1.17	1.122
	[0.936 - 1.545]	[0.921 - 1.534]	[0.836 - 1.793]	[0.821 - 1.773]	[0.954 - 1.493]	[0.948 - 1.499]	[0.937 - 1.461]	[0.893 - 1.410]
Salary	1.408***	1.387***	0.927*	0.909*	1.692***	1.688***	2.586***	2.453***
	[1.299 - 1.527]	[1.253 - 1.535]	[0.852 - 1.009]	[0.816 - 1.011]	[1.560 - 1.835]	[1.524 - 1.870]	[2.381 - 2.809]	[2.211 - 2.723]
Salaried * high layoff plants		1.041		1.054		1.006		1.146
		[0.885 - 1.224]		[0.890 - 1.248]		[0.856 - 1.182]		[0.972 - 1.351]
Observations	14079	14079	14072	14072	14055	14055	14059	14059
Number of locations	30	30	30	30	30	30	30	30

95% Confidence intervals are in brackets.

Asterisk indicate significance level of estimated coefficient: *** p < 0.01, ** p < 0.05, * p < 0.1.

Note: Both model 1 & 2 include controls for worker’s tenure, worker’s race, worker’s gender, whether the worker was employed at a union plant, and the change in the prior year’s unemployment rate in the country of the plant. Model 2 incudes an additional interaction term between working at a high layoff plant and worker type to allow for a differential effect of working at a high layoff plant by worker type on stress outcomes.

Though reports of work stress were much lower in 2006 overall (Figure 2), salaried workers reported more work stress even in the 2006 survey. Salaried worker reported significantly higher work stress for two of the three work stress questions, I find my work stressful (OR = 1.41; 95% CI 1.30- 1.53) and I find that work issues frequently remain on my mind after hours (OR = 1.69; 95% CI 1.52 – 1.87). They were also somewhat less likely to report being worn out at the end of the day compared to hourly workers (OR = 0.91; 95% CI 0.81-1.01). There was no difference in reports of work stress or workers’ pride in Alcoa for workers who worked at plants that would have high layoffs in 2009.

## Discussion

This study explored the consequences of downsizing on work stress for survivors of layoffs in the context of a severe recession at a large multi-site manufacturing company. The results show that salaried workers consistently report more work stress, but that by 2010 –one year after severe downsizing– salaried workers at plants with high layoffs reported that they experienced more work stress than their counterparts at non-high layoff plants. The differential increase in work stress was found in two of the three work questions, and was corroborated in all three independent surveys done in 2010, 2011, and 2012. In contrast, though we documented more variation over time in the control question we examined, we did not observe any difference in reports of worker pride in Alcoa in high layoff plants further corroborating that our results were not an artifact. We also showed that there were no pre-existing differences in reports of work stress or pride in Alcoa at plants that would eventually have high layoffs in 2009.

The results presented here are also in line with previous studies. A recent study using a population sample showed that among middle-aged, college-educated white men with full-time employment report increased stress from 2006 to 2009 [19]. This population would be very similar to the group that reported increased stress in our sample. In a more direct comparison, previous work in this workforces showed that employees who survive layoff but work at plants with severe downsizing, experience a higher incidence rate for hypertension than their counterparts in less affected plants [17]. Given the previously reported relations between work stress and health broadly, and previous findings within this workforce that remaining workers in high layoff plant after the 2009 layoffs had worse health outcome, we hypothesize that work stress may be a pathway linking the external economic forces with the health status change. In order to test such a hypothesis, that work stress mediated the adverse health outcomes previously found, we would need to examine whether including an individual’s work stress would eliminate any association between health and working in a high layoff plant. However, we could not link individuals in the work engagement surveys to their health claims data, which was used in the previous analysis, to conduct a full mediation study.

Despite the inability to conduct mediation analyses, this study has several notable strengths. First we find convergence with regard to work-stress from three independent samples of the same workforce, which bolsters our confidence in the result. Second, we do not find that salaried workers at high layoff plants report more stress in either 2006 or 2009, suggesting that our results are not due to pre-existing plant differences. Third, our examination of the control question suggests that while some workers may have reported positive responses to please management, this practice was not systematic in high layoff plants. Had we found that workers in high layoff plant reported differential pride in working at Alcoa our interpretation of the results — that downsizing leads to higher work stress— would be undermined. Fourth, even though we are examining only one organization, the 30 plants studied cover 16 states and all census regions, suggesting that our results are unlikely due to localized effects.

The interpretation of results of this study in relation to previous studies of work stress, most notably from the cohort of civil servants in Whitehall II study [20], require an understanding of the differences in the aim and context of the current study. The present study aims to assess changes in workers’ reported stress to a firm-level exogenous stressor, severe downsizing, as opposed to examining the role of a specific job on work stress. The finding that salaried workers within the manufacturing sector reported higher levels of stress after the layoffs, while initially unexpected, are consistent in the context of the study population for several reasons. First, salaried workers reported more work stress even before the recession, suggesting that even in normal times salaried workers bear more stress in this context. Second, we find evidence of increases in reported stress in high layoff plants for salaried workers in 2010–2012, after the 2009 layoffs. According to BLS data, productivity grew by 6% in 2010 for the manufacturing sector as a whole, yet we can imagine that in certain hourly jobs (assembly line, pot rooms, etc.) there is little room for speed up and increased productivity gains, whereas there is more leeway for productivity gains for administrative and technical jobs in the salaried portion of the workforce. It is likely that during downsizing salaried workers are more vulnerable to demands of increased effort or intensity without compensating rewards because hourly workers have union protection regulating any possible speed up and because any increase in work hours is directly compensated at the known hourly rate or through overtime. Hourly workers know their exact compensation for their efforts, whereas salaried workers may experience more effort-reward imbalance because their compensation is not directly tied to their efforts, especially in the short-run. Furthermore, salaried workers were at risk of having more uncompensated work demands and/or having the scope of their work duties expanded after the recession. Hourly workers were protected from such changes in scope of their work and any extra effort would have been compensated. Thus, the increase in work stress may be concentrated in the salaried part of the workforce because of increased workloads and job insecurity. While we cannot directly observe differential changes in work demands, there was some rehiring of hourly workers in 2010–2012, whereas staffing levels remained depressed in the salaried workforce.

## Conclusion

The negative consequences of the recent recession among workers who have not themselves been displaced, but have experienced severe layoffs in their own firms have been underexplored. This study is an attempt to explore work stress as a proximal “outcome” for this population. Our results suggest that downsizing led to persistent work stress, which accumulated overtime and may in turn lead to other negative outcomes, particularly negative health outcomes as have been found previously [2-4,10-14,21]. Increased work stress is important to monitor and may be a mediating pathway through which the external economic environment leads to adverse health outcomes. Future studies will need to explore the links between persistent stress and health consequences after downsizing events.

## Abbreviations

CI: Confidence interval; OR: Odds ratio.

## Competing interest

Authors have no competing interest to declare. Dr. Cullen serves as a senior medical advisor to Alcoa under the terms of a research contract between Stanford University and Alcoa, Inc.

## Authors’ contributions

SM MC conceived of study. SM did data analysis. SM structured and wrote the manuscript. MC participated in the presentation of results and contributed to writing the manuscript. Both authors read and approved the final manuscript.

## Pre-publication history

The pre-publication history for this paper can be accessed here:

http://www.biomedcentral.com/1471-2458/13/929/prepub
